# Colorimetric CO_2_ Indicators

**DOI:** 10.1021/accountsmr.2c00226

**Published:** 2023-06-19

**Authors:** Andrew Mills, Lauren McDonnell, Dilidaer Yusufu

**Affiliations:** School of Chemistry and Chemical Engineering, Queens University Belfast, David Keir Building, Stranmillis Road, Belfast BT95AG, U.K.

## Abstract

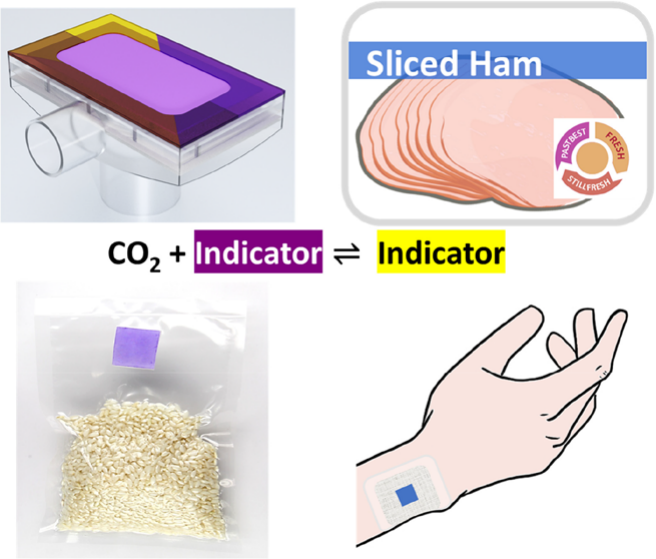

Carbon dioxide, CO_2_, is an essential part of life, in that through green plant photosynthesis it is used to generate food and fuel and is generated in both aerobic and anaerobic respiration. Industrially, it is used in fire extinguishers, supercritical fluid extractions, and food packaging. Environmentally, it is in the atmosphere, hydrosphere, and biosphere and is responsible for global warming and the acidification of the oceans. The monitoring of CO_2_ in the gas phase is usually carried out using FTIR spectroscopy, whereas the measurement of dissolved CO_2_ usually involves an electrochemical device. Excitingly, the most recent forms of CO_2_ indicators appear to offer significant advantages over current methods, such as simplicity, low cost, and portability.

This Account highlights the work of the Mills group on transforming CO_2_ colorimetric indicator technology from the usual water-based (i.e., “wet”) indicator form to dry CO_2_-sensitive inks, pigments, plastics, and adhesives. Initially, the basic theory associated with colorimetric CO_2_ indicators is described, and the simple relationship between indicator absorbance and the partial pressure of CO_2_, *P*_CO2_, established. The early work on CO_2_-sensitive inks is then described, where such inks comprise a hydrophilic pH-sensitive dye anion, coupled with a lipophilic quaternary ammonium cation, dissolved in a nonaqueous solution of a polymer which, when cast, forms a dry ink film that gives a reversible color response when exposed to CO_2_ both in the gas phase and dissolved in solution. The ability to tune the sensitivity of a CO_2_ ink film to the desired application through the judicious choice of the pH indicator dye and base concentration is described. The dependence of the sensitivity of a CO_2_ ink film on temperature is used to create a temperature indicator, and the ability to tune the ink, to respond to high levels of CO_2_, is used to create a fizziness indicator for carbonated drinks. Very sensitive CO_2_ inks are used to make a vacuum and a general air-pressure indicator. The more recent development in CO_2_ indicator technology is described in which CO_2_ inks are used to coat silica particles to make a range of different CO_2_-sensitive pigments, which, when incorporated into a plastic, through extrusion, produce a range of novel CO_2_-sensitive plastic films that have many notable advantages over their ink film counterparts. Examples are then given of such plastic films being used for dissolved CO_2_ measurements in salt water, for food packaging, and as an early wound-infection indicator. Finally, the recent incorporation of a CO_2_-sensitive pigment into a pressure sensitive adhesive to make an after opening freshness tape is described briefly.

Although most commercial CO_2_ indicators are assessed by eye and so are limited to qualitative analysis, this work shows that colorimetric CO_2_ indicators can be used for quantitative analysis through absorbance measurements. Nowadays, such measurements can be readily made using just a digital camera and color analysis software via digital camera colorimetry, DCC, which is likely to have a significant impact on the widespread use of the CO_2_ indicators described herein, their commercial viability, and their potential areas of application.

## 1 Introduction

Carbon dioxide, CO_2_, is a basic chemical feedstock of life, as it is used to generate the fuel and food necessary for most life forms and is a common indicator of life and health. Although, there is little CO_2_ in the atmosphere, ca. 412 ppmv (0.04%), it is rising and so creating environmental problems such as global warming and the acidification of the oceans.^1,2^ Thus, the monitoring of the levels of CO_2_ in the atmosphere, hydrosphere, and biosphere is a core part of environmental analysis.^3^ In industry, CO_2_ is used as an inert gas in welding and fire extinguishers, as a pressurizing gas in oil recovery, and as a supercritical solvent in the decaffeination of coffee and supercritical drying.^4^ In the drinks industry, CO_2_ is used to make a myriad of carbonated beverages and, in the food industry, as an active packaging gas, since it has antimicrobial activity. Its use in modified atmosphere packaging, MAP, accounts for over 60 billion food packages per annum.^5^ In medicine, the measurement of dissolved CO_2_ levels in blood and the monitoring of the CO_2_ in breath, i.e., capnography, are routine.^6,7^

In recent years, a number of color-based CO_2_ indicators have emerged as possible, inexpensive, disposable alternatives^8,9^ to the usual bulky, expensive methods used to analyze for CO_2_ in air or dissolved in solution, such as infrared spectroscopy and the Severinghaus electrode, respectively.^10,11^ This Account outlines the recent evolution of CO_2_ colorimetric indicators, from inks to pigments, plastic films, and adhesives, that has been pioneered by the Mills group. This is not to say other groups have not contributed to the development of such indicators,^12−18^ and a list of some of the major advances made by such groups over the years is given in Table S1 in the Supporting Information, SI, file that accompanies this paper.

There have been several major reviews on CO_2_ indicators, and most have focused on their use in food packaging.^19−21^ However, this review is different in that, through the work of one group, the evolution of CO_2_ indicators over the last 3 decades is described, with particular attention to how they work and potential applications other than in food packaging.

## 2 Optical CO_2_ Indicators Theory

Most, if not all, color-based CO_2_ indicators use a pH indicator dye, D, which responds to the change in pH in the surrounding (encapsulating) medium, be it an aqueous solution or an ink film, due to the formation of carbonic acid and its subsequent deprotonation. The latter process can be summarized by the following reaction,
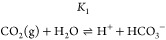
1In most, if not all, CO_2_ indicators, sufficient base is present so that the partial pressure of CO_2_, *P*_CO2_, is related directly to the concentration of H^+^, [H^+^], i.e.,
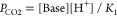
2where [Base] is the base concentration, which is usually sodium bicarbonate, NaHCO_3_, in aqueous solution, and a quaternary ammonium hydroxide, Q^+^OH^–^·*x*H_2_O, in the case of CO_2_-sensitive inks, pigments, and films. When a pH indicating dye is also present, as with a CO_2_ colorimetric indicator, the following equilibrium is also set up,
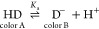
3where *K*_a_ is the acid dissociation constant of the pH indicator dye and HD and D^–^ are the protonated and deprotonated forms of the dye, with colors A and B, respectively. The dye concentration is very small and, usually, has no significant effect on the equilibrium reaction 1, and so the following expression can be derived, relating the color of the indicator to *P*_CO2_,
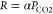
4where *R* is the ratio of the concentrations of HD and D^–^, i.e., [HD]/[D^–^], α is a proportionality constant (= *K*_1_/*K*_a_[Base]), and *P*_CO2_ is the partial pressure of CO_2_, which here, as is common practice, is expressed as a percentage of an atmosphere, where *x*% CO_2_ = 0.01·*x* atm CO_2_. Equation 4 is found to apply to most colorimetric CO_2_ indicators. From eq 4 it follows that at *R* = 1, the indicator is halfway through its color change, and the associated level of *P*_CO2_, *P*_CO2_(S = 1/2), is equal to 1/α.

In order to use a CO_2_ indicator for quantitative analysis, usually its absorbance, *A,* is monitored at a wavelength where D^–^ absorbs strongly, and in this work, in all the examples cited, *A* is the absorbance of the indicator at the maximum absorbance wavelength associated with the deprotonated form of the dye, D^–^. Under such conditions, *A* is related to *R,* and so *P*_CO2_ by the following expression,
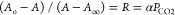
5where *A*_o_ and *A*_*∞*_ are the fixed, measured absorbances of the CO_2_ indicator when the dye is completely in its deprotonated and protonated form, respectively, i.e., in its extreme color forms, color B and color A, respectively. In practice, the values of *A*_o_ and *A*_*∞*_ are usually taken as the measured values of *A* when the indicator is exposed to the extreme levels of CO_2_, of 0 and 1 atm, respectively.

## 3 CO_2_ Indicator Ink Films

Up until the early 1990s, work on CO_2_ indicators had been limited to systems in which a pH indicator dye was dissolved in an aqueous solution. Examples include the drop-checker CO_2_ indicator used in aquaria^22,23^ and the “Einstein” indicator for ensuring correct tracheal intubation.^12^ When such an indicator is used to measure the level of dissolved CO_2_ in a test medium, the CO_2_-sensitive aqueous indicator layer has to be confined behind a thin polymer film, such as polyethylene terephthalate, PTFE, which acts as a waterproof, gas-permeable (ion-impermeable) membrane, i.e., a GPM.^14,24^

In the early 1990s, the Mills group were the first to report a “dry” CO_2_ indicator film, created using a solvent-based ink^25,26^ in which the highly hydrophilic anionic form of a pH indicator dye was rendered solvent-soluble by pairing it with the quaternary cation, Q^+^, of a phase transfer agent, PTA, Q^+^OH^–^·*x*H_2_O,
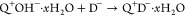
6where the ion pair, Q^+^D^–^·*x*H_2_O, had the added attractive feature of possessing a few molecules of water of hydration even in a nonaqueous, lipophilic medium.^27^ The above reaction allowed D^–^ to be dissolved in the same lipophilic solvent as a hydrophobic polymer, such as ethyl cellulose, EC, to form a solvent-based ink. On casting the ink onto an inert substrate and allowing it to dry, a thin, hydrophobic, water insoluble, CO_2_ indicator ink film is created, which gives a reversible color response to CO_2_,
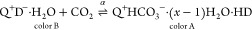
7 where Q^+^D^–^·*x*H_2_O and Q^+^HCO_3_^–^·(*x* – 1)H_2_O·HD are the lipophilic, deprotonated, and protonated ion-paired forms of pH indicator dye.^25,26^ It follows from eq 7 that the absorbance of the film, due to Q^+^D^–^·*x*H_2_O, *A*, will be related to *P*_CO2_ as described by eq 5, where α is the equilibrium constant for reaction 7 and R = [Q^+^HCO_3_^–^·(*x* – 1)H_2_O·HD]/[Q^+^D^–^·*x*H_2_O].

**Figure 1:**
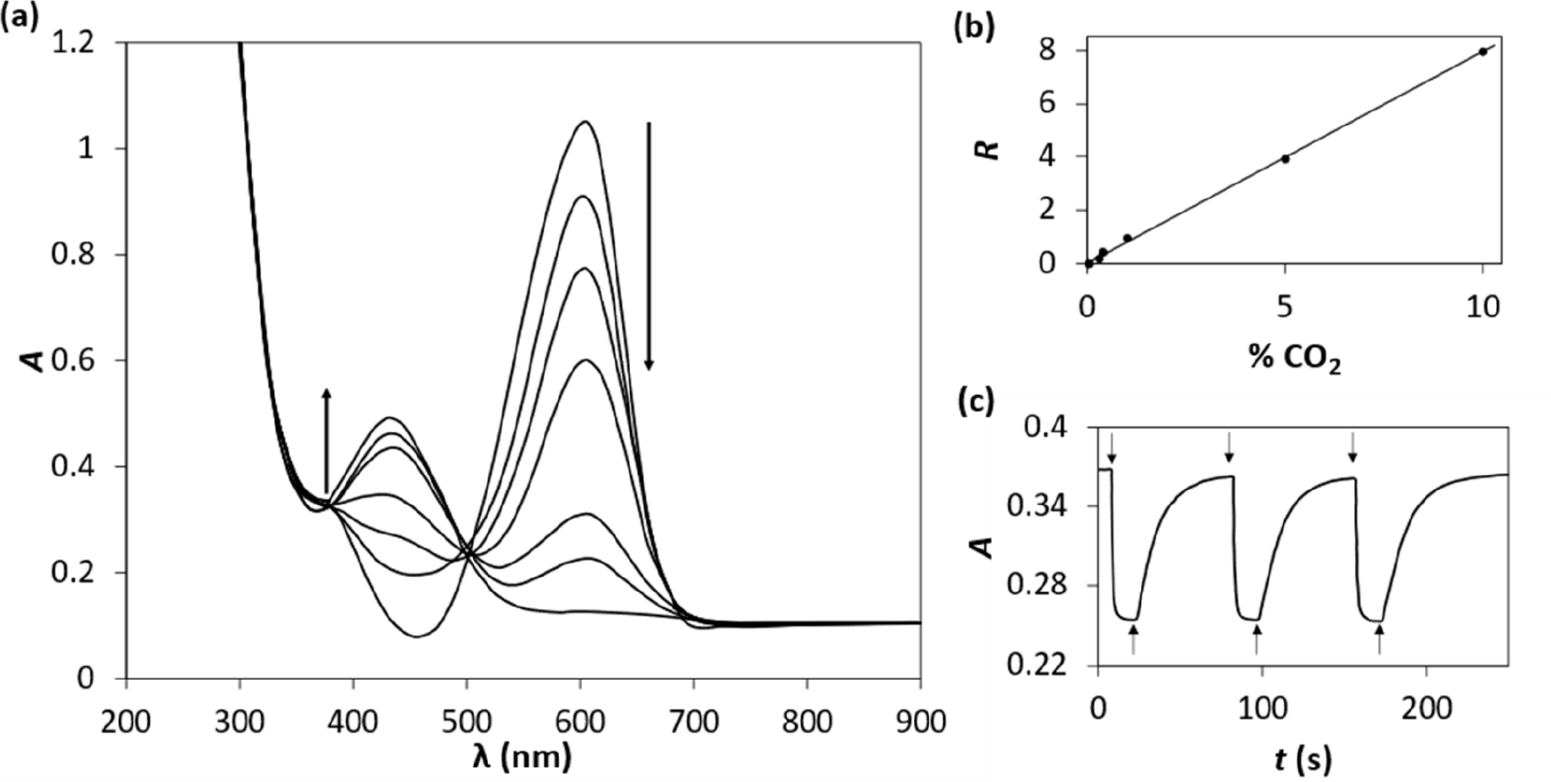
(a) UV/vis absorption spectra of a MCP ink film recorded when exposed to different CO_2_/N_2_ gas mixtures, with the % CO_2_ (from top to bottom) being 0.04, 0.3, 0.4, 1.0, 5.0, 10 and 100%, respectively, with the peaks at 605 and 420 nm, decreasing and increasing, with increasing % CO_2_, respectively; (b) plot of *R* vs % CO_2_ for the MCP indicator, where *R* was calculated using eq 5 and values of *A* taken from (a); (c) *A* vs time plots for the MCP indicator ink film on exposure to an alternating gas supply of air, ↑, and 5% CO_2_, ↓. [MCP/EC/TOAH/TBP film; *T* = 20 °C; gas flow rate, *f*, 100 cm^3^/min; relative humidity, RH, 0%.] Adapted from ref (26). Copyright 1992 American Chemical Society.

One of the first reported CO_2_-sensitive ink films comprised the deprotonated form of the pH indicating dye, *meta*-cresol purple, MCP, ion-paired with a tetraoctyl ammonium quaternary cation, from the lipophilic base, tetraoctyl ammonium hydroxide, TOAH, dissolved in a nonaqueous solvent in which were also dissolved the lipophilic polymer, EC, and a plasticizer, tributyl phosphate, TBP. An abbreviated formulation of this ink film is, therefore, MCP/EC/TOAH/TBP, i.e., dye, polymer, PTA, and plasticizer, and is used in the caption in Figure 1, along with other important test condition details. In this and all other inks described herein, the polymer (EC) was used to provide a lipophilic medium to dissolve the ion-pairs formed between the dye (MCP) and the PTA (TOAH) when the ink had been cast as a film and the solvent had evaporated. The plasticizer (TBP) of the polymer (EC) was used to improve the rate of permeation of CO_2_ through the dried ink film.^28,29^ The resulting MCP CO_2_ indicator ink film was purple or yellow in the absence or presence of CO_2_, respectively. A brief experimental detailing how the MCP ink film was made is given in section S2.1 of the SI file.

Figure 1a illustrates the UV/vis absorption spectral changes exhibited by the MCP ink film when exposed to different % CO_2_ levels. From the data in Figure 1a, the absorbance of this film, *A,* was determined as a function of % CO_2_, which, via eq 5, was then used to generate the plot of *R* vs % CO_2_ illustrated in Figure 1b. The straight-line nature of this plot is as predicted by eq 5, from the gradient of which values of α = 0.78% CO_2_^–1^ and *P*_CO2_(S = 1/2) value (= 1/α) of 1.3% CO_2_, were calculated.

Another key characteristic of a CO_2_ indicator is its 90% response and recovery time, *t*_90↓_ and *t*_90↑_, respectively. In order to measure the latter, the absorbance of the MCP ink film was monitored as a function of time upon exposure to an alternating atmosphere of 0.04% (i.e., air) and 5% CO_2_, the results of which are illustrated in Figure 1c and reveal *t*_90↓_ and *t*_90↑_ times of 2.6 and 31 s, respectively;^26^ further practical details are given in S3 of the SI. Although the above *t*_90↓_ and *t*_90↑_ values are not particularly large, they prevent the indicator from being used for certain applications, such as in capnography, which requires response/recovery times of ca. ≪1 s.^30^

The response and recovery times of this, and most CO_2_ indicator films, depend simply upon the rate of diffusion of the CO_2_ into and out of the indicator film, and not the kinetics of reaction 7.^31^ Consequently, they can be markedly shortened by making the films thinner or increasing the operating temperature.

### 3.1 Dependence of Sensitivity (α) on Dye p*K*_a_ and [Base]

As noted earlier, the sensitivity of the CO_2_ ink film indicator depends directly upon the value of α, i.e., the gradient of the *R* vs % CO_2_ plot, where, according to eq 4, α is inversely proportional to *K*_a_ and [Base]. Thus, early on in the development of CO_2_-sensitive ink films, the variation in *P*_CO2_(S = 1/2), = 1/α, was studied as a function of both *K*_a_ and [Base].^32^ In the former case, a number of different dyes, with different p*K*_a_ values, were used, namely, rosolic acid (RA), phenol red (PR), cresol red (CR), MCP, thymol blue (TB), ortho-cresol phthalein (OCP), and phenolphthalein (PP). A list of these dyes, their abbreviated names, p*K*_a_ values, and colors in their Q^+^D^–^·*x*H_2_O and Q^+^HCO_3_^–^·(*x* – 1)H_2_O forms are given in section S4, Table S2 in the SI. The different ink films were formulated as described for the MCP ink film in section S2.1 in the SI, with the only altered parameter being the pH dye used. For each film, the associated value of *P*_CO2_(S = 1/2) was determined from a *R* vs % CO_2_ plot using the same method as described above for the MCP ink film and illustrated by the results in Figure 1a and b.

**Figure 2:**
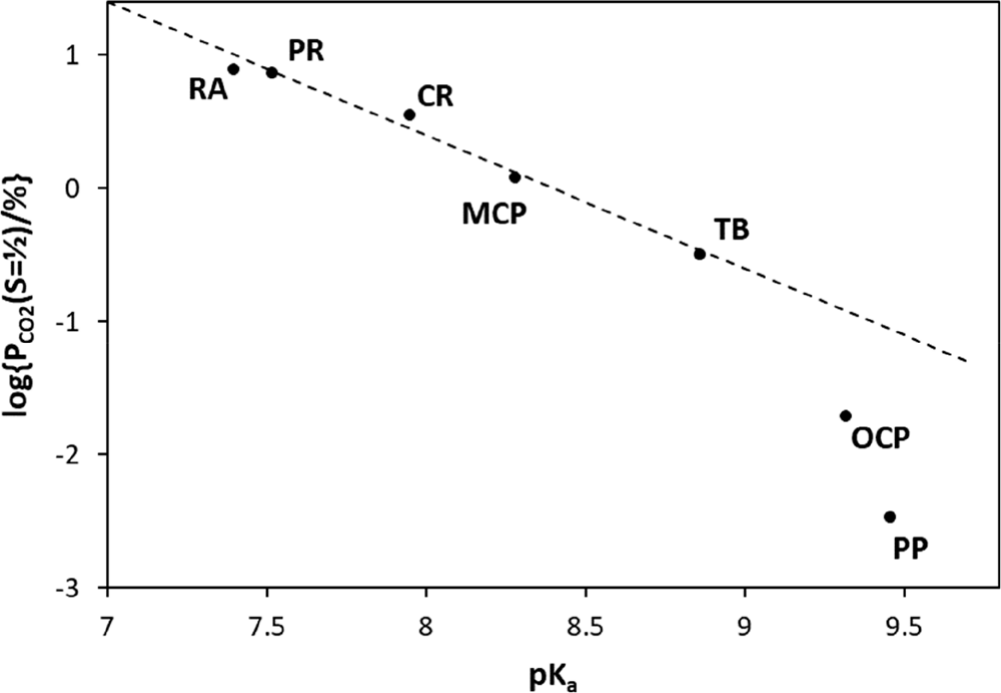
Plot of log{*P*_CO2_(S = 1/2)} vs p*K*_a_, where the values of {*P*_CO2_(S = 1/2)} were determined from *R* vs % CO_2_ plots of the absorbance data derived from CO_2_ indicator ink films employing the following, different pH indicator dyes, rosolic acid (RA), phenol red (PR), cresol red (CR), MCP, thymol blue (TB), ortho-cresol phthalein (OCP), and phenolphthalein (PP). [dye/EC/TOAH/TBP films; *T* = 20 °C; *f*, = 100 cm^3^/min, RH = 0%.] Adapted with permission from ref (32). Copyright 1994 Elsevier.

The plot of the results of this work, in the form of log{*P*_CO2_(S = 1/2)} vs p*K*_a_, is illustrated in Figure 2 and shows that, in accord with eq 4, the sensitivity (α) increases, and so *P*_CO2_(S = 1/2) decreases, with a decreasing value of *K*_a_ (increasing value of p*K*_a_). Equation 4 also predicts that a plot of log{*P*_CO2_(S = 1/2)} vs p*K*_a_ should be a straight line with a gradient, *m*, of −1, which is represented by the broken line in Figure 2, from which it appears that this relationship only holds over the p*K*_a_ range 7–9, with dyes OCP and PP, and their >9 p*K*_a_ values, appearing as notable exceptions. The same deviation from linearity is also seen for the same dyes in aqueous solution^32^ and is due to the breakdown of the underlying assumption in eq 4, that the deprotonation of the bicarbonate to carbonate is not significant, as its p*K*_a_ is ca. 10.3. This assumption is not valid at very low %CO_2_ values, and so *m* tends to −2, which helps explain the apparent steep departure from the broken line (*m* = −1) in Figure 2 exhibited by OCP and PP.^32^

In other work, the dependence of α on 1/[Base], also predicted by eq 4, has been shown to hold for CO_2_ indicator ink films.^32,33^

### 3.2 Dependence of Sensitivity upon Temperature

**Figure 3:**
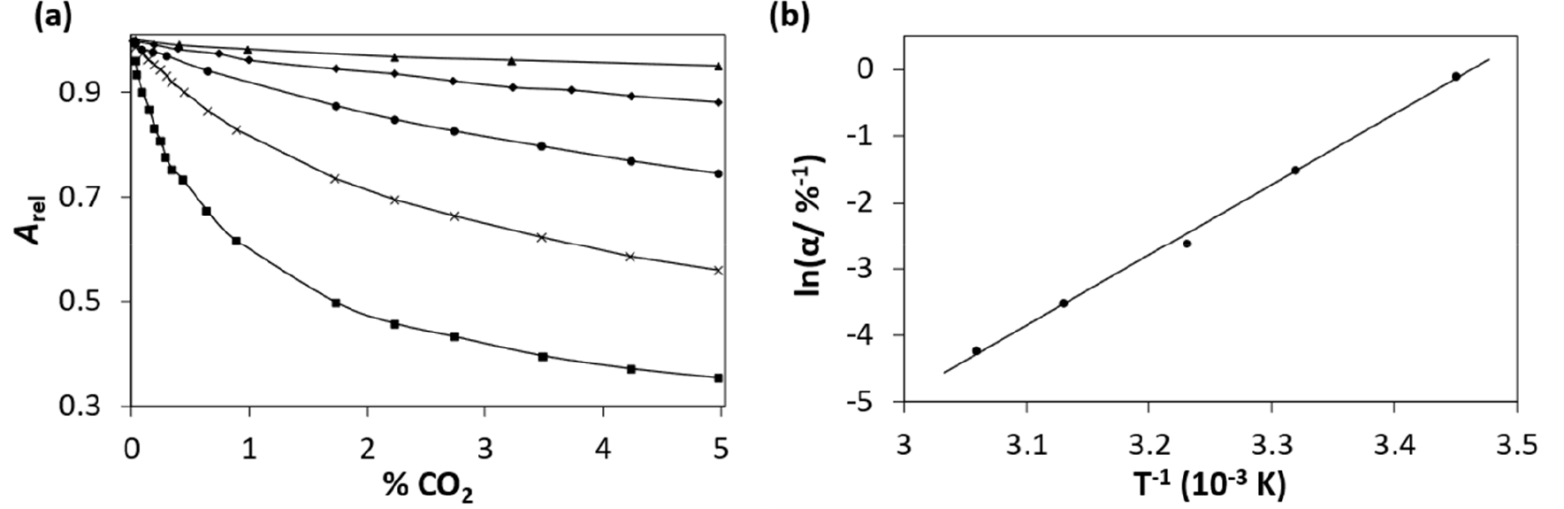
(a) Relative absorbance, *A*_rel_ (at λ(max) for D^–^), as a function of % CO_2_ for a PR ink film, recorded at the following temperatures (from bottom to top): 17, 28, 37, 46, and 55 °C, respectively; (b) plot of the values of ln(α), derived from the data and eq 5, in (a), vs 1/*T*. [PR/EC/TOAH/TBP films; *T* = 20 °C; *f* = 100 cm^3^/min, RH = 0%.] Adapted with permission from ref (30). Copyright 1997 Elsevier.

All CO_2_ colorimetric indicators, i.e., water-based, indicator ink, smart pigment, and plastic films, exhibit a decreasing sensitivity, α, with increasing temperature, implying an overall exothermic process, most likely linked to the dissolution of CO_2_ in the encapsulation medium, which is known to be exothermic in both aqueous and nonaqueous solutions.^34^Figure 3a provides a suitable illustration of this dependence for a PR ink film, in the form of a plot of the measured absorbance of the film, due to Q^+^PR^–^·*x*H_2_O as a function of % CO_2_, recorded at different temperatures.^30^ The data associated with each *A* vs % CO_2_ profile in Figure 3a were used to generate a *R* vs % CO_2_ plot, using eq 5, from which a value of α was derived. This data was then used to construct the plot of the ln(α) vs 1/*T* illustrated in Figure 3b, which fitted the basic thermodynamic expression,
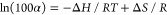
8where the product, 100α, is the equilibrium constant for reaction 5 in units of atm^–1^. From the plot in Figure 3b and eq 8, values of ca. −88 kJ mol^–1^ and −266 J mol^–1^ K^–1^ were calculated for Δ*H* and Δ*S*, respectively, for reaction 5 values which are consistent with those reported for other ink films^26^ and with reaction 7 and its expected exothermicity.

It follows from the above discussion that a CO_2_ indicator film could be used as a temperature indicator, if the value of *P*_CO2_ was set at some fixed value, *P*_CO2_(fxd), and details of such a study are given in section S5 of the SI and ref (35).

### 3.3 Applications

The above work shows clearly that CO_2_ ink films can be used to provide quantitative information regarding the level of CO_2_ in the ambient gas phase. Subsequent studies were then carried out to explore their potential areas of application, the details of which are given below.

#### 3.3.1 Capnography

**Figure 4:**
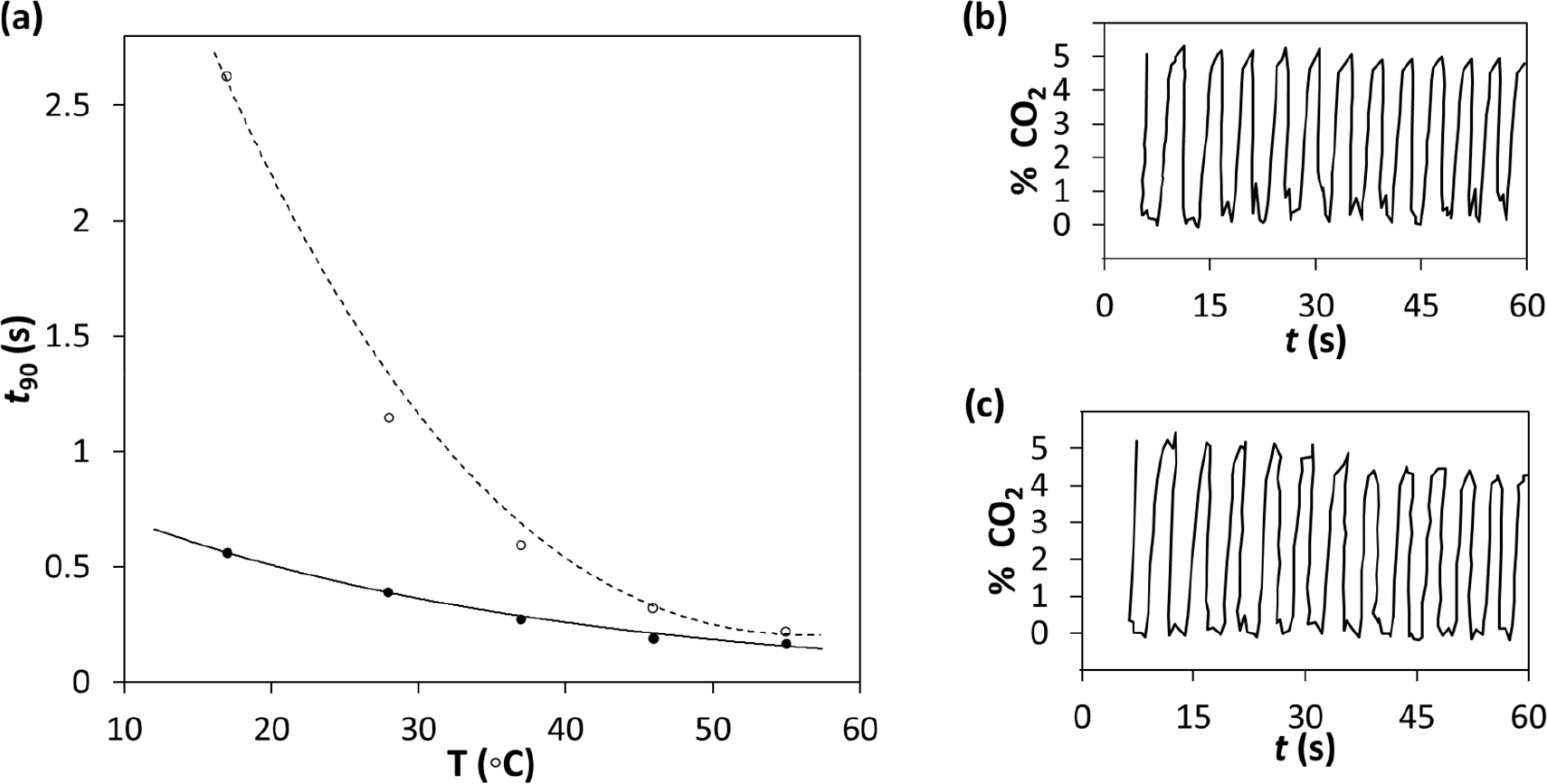
(a) Plot of the measured *t*_90_ values for response (solid line) and recovery (broken line) times for a PR ink film when exposed to an alternating atmosphere of 0 and 5% CO_2_, as a function of indicator film temperature; % CO_2_ vs time real breath profiles recorded using (b) the PR ink film and (c) a commercial capnometer. [PR/EC/TOAH/TBP films; *T* = 20 °C; *f* = 100 cm^3^/min, RH = 0%.] Adapted with permission from ref (30). Copyright 1997 Elsevier.

In section 3, it was noted that, for a MCP ink film, although only a few seconds, the *t*_90↓_ and *t*_90↑_ values were still too big for monitoring CO_2_ accurately in breath, i.e., capnography, which requires response/recovery times of ca. ≪1 s.^30^ However, subsequent work on a PR ink film showed that both *t*_90↓_ and *t*_90↑_ decreased markedly with increasing temperature (see Figure 4a) and this feature allowed the indicator, when operated at 50 °C, to produce % CO_2_ versus time profiles for real breath cycles that were near-identical to those recorded by a commercial capnometer, as illustrated in Figure 4b and c, respectively.^30^

#### 3.3.2 Quality Control of Carbonated Drinks

One potential area of application of a CO_2_ indicator is in the measurement of the high *P*_CO2_ in a carbonated drink, i.e., as a fizziness quality control indicator. The production of carbonated beverages is a billion-dollar industry in which it is essential that every bottle contains the appropriate high level of CO_2_, typically ca. 4 bar. However, at present, there is no simple, inexpensive method to measure the *P*_CO2_ in carbonated drink bottles.

A fizziness indicator would benefit not only the packager but also the retailer and consumer, as it would flag leaky bottles on the supermarket shelf and inform the consumer if a previously opened bottle has lost its sparkle, i.e., is flat. Thus, a fizziness indicator was developed based on a water-based ink with PR, sodium hydroxide, poly(vinyl alcohol), PVA, and glycerol, as the pH indicating dye, base, polymer, and plasticizer, respectively.^36^ A water-based ink was selected because, when using the same dye, such inks are significantly less sensitive than their solvent ink counterparts, most likely due to the much lower solubility of CO_2_ in water (and the hydrophilic polymer PVA) than in organic solvents (and hydrophobic polymers, such as EC).

**Figure 5:**
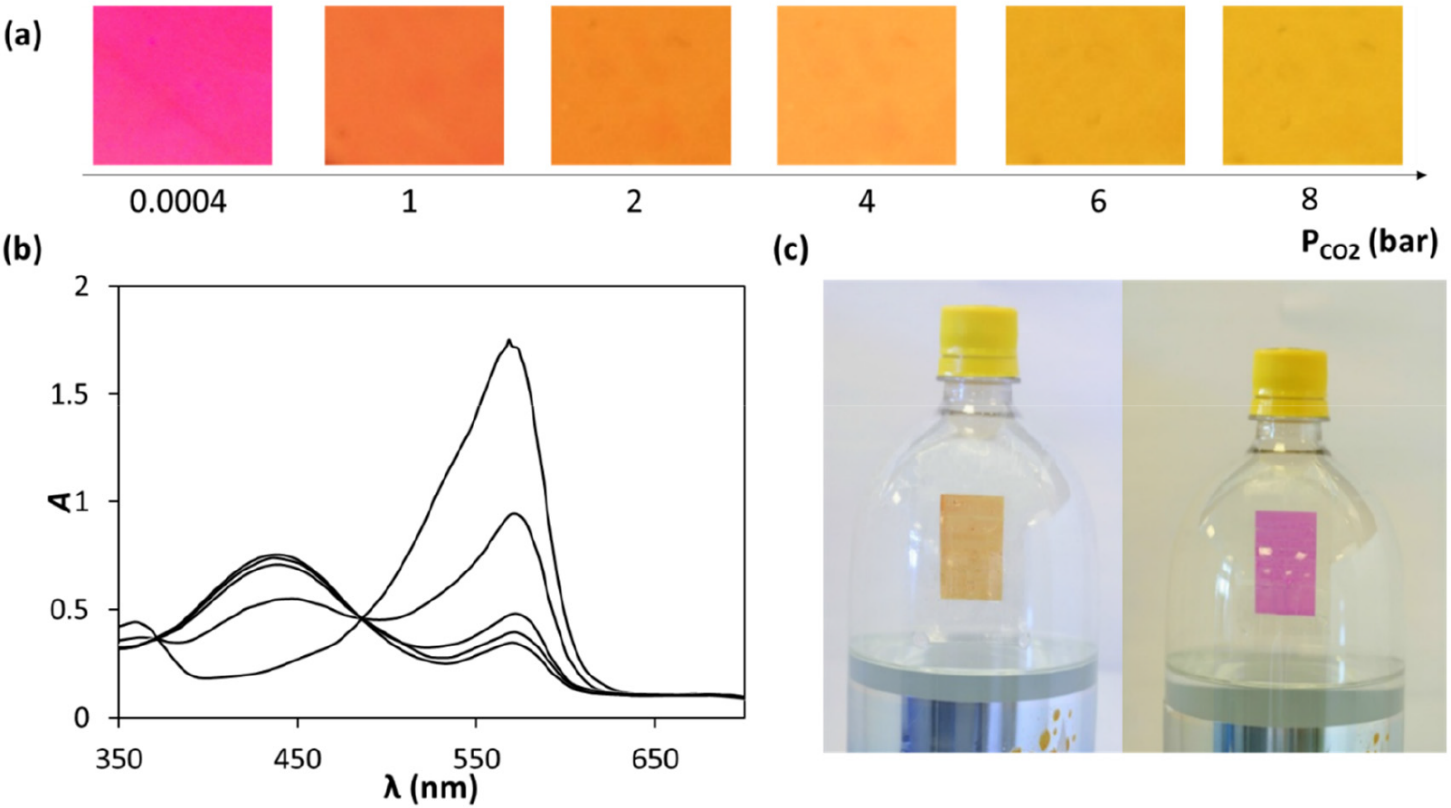
(a) Photographs of a PR/NaOH/PVA/glycerol water-based CO_2_ indicator film and (b) spectra (from top to bottom) as a function of increasing *P*_CO2_; (c) photographs of (from left to right) a fizzy and flat carbonated drink. [PR/PVA/NaOH/films; *T* = 20 °C; *f* = 0 cm^3^/min, RH = 0%.] Adapted with permission from ref (36). Copyright 2011 Royal Society of Chemistry.

For example, a typical solvent-based PR ink film has a *P*_CO2_(S = 1/2) value of 7.4% (see Figure 2), whereas for a water-based PR ink film it is ca. 150%, i.e., ca. 1.54 bar!^36^ The colors and spectra of this fizziness indicator, when exposed to different high *P*_CO2_ values, are illustrated in Figure 5a and b. Figure 5c shows the fizziness indicator in a carbonated drink bottle when the liquid was either fully carbonated or flat.^36^ Although of promise, since the ink used in this work is water-based, dye leaching is a problem, and so, in practice, in a carbonated drink bottle, it would need to be laminated with a GPM to prevent dye leaching or replaced with a plastic film equivalent, vide infra.

#### 3.3.3 Manometry

An ink film that is sufficiently sensitive to respond to 0.04% CO_2_ can function as an air pressure, *P*_air_, sensor, provided the temperature is fixed since the value of *P*_CO2_ is directly proportional to *P*_air_. One area of possible application is in vacuum packaging (VP), which is commonly used in wholesale and retail food packaging, since there is no simple, inexpensive method for measuring the vacuum pressure inside such packages.

**Figure 6:**
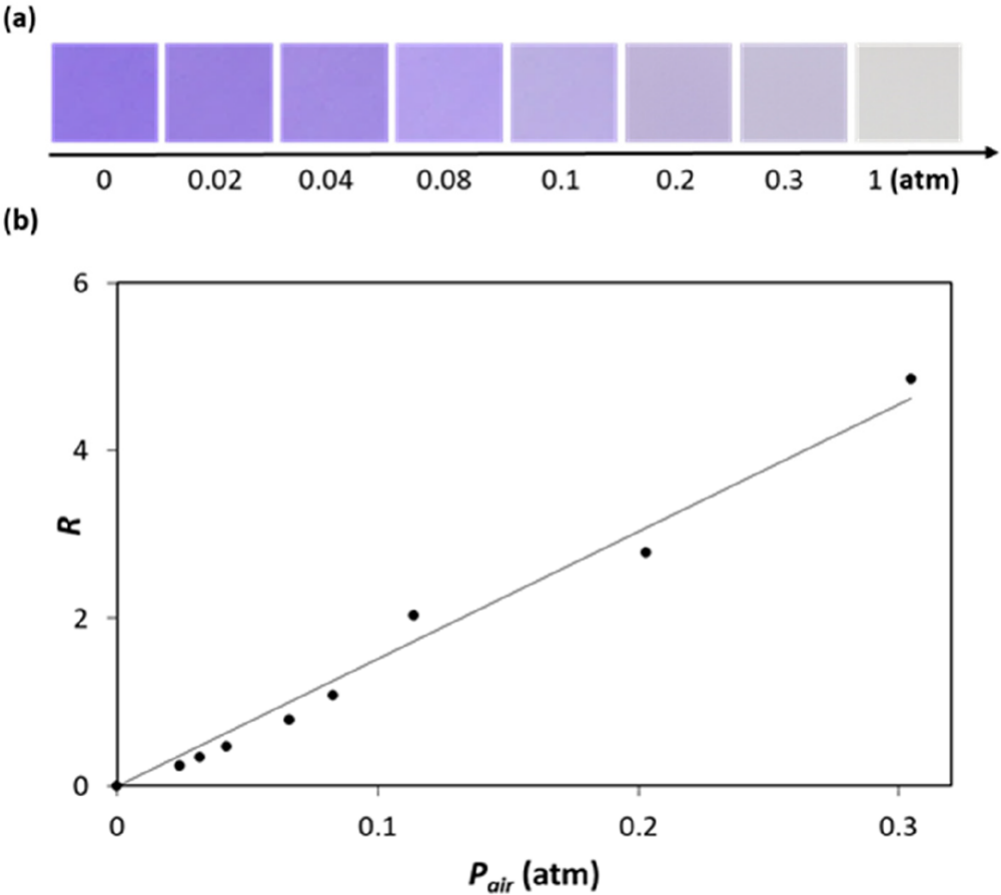
(a) Photographs of an OCP CO_2_ indicator at different vacuum air pressures, *P*_air_, at 22 °C, and (b) subsequent plot of the associated absorbance data in the form of *R*, calculated using eq 5, vs *P*_air_, with a line of best fit of gradient 16.1 atm^–1^. [OCP/EC/TBAH/TBP films; *T* = 22 °C; *f* = 0 cm^3^/min, RH = 0%.] Adapted with permission from ref (37). Copyright 2019 Royal Society of Chemistry.

A vacuum pressure indicator, based on a CO_2_-sensitive OCP ink, has been reported,^37^ and Figure 6a illustrates the observed variation in color exhibited by the OCP ink film as a function of air pressure over the range 0–1 atm at 22 °C. From the measured absorbance, *A*, of the film recorded at different *P*_air_,, the plot of *R* vs *P*_air_, illustrated in Figure 6b, was generated, from which a value of *P*_air_ at which the OCP indicator film is halfway through its color change, i.e., P(S = 1/2), of ca. 0.62 atm was derived. Given the vacuum pressure in a typical VP food product is ca. 0.04 atm,^38^ the above *P*_air_(S = 1/2) value of ca. 0.62 atm determined for the OCP indicator shows it is well-suited for monitoring the pressure inside VP products.

The OCP CO_2_ indicator ink film is appropriate for monitoring air pressure values well below 1 atm but not for monitoring levels at or above 1 atm. Thus, in a subsequent study a general-purpose air pressure indicator was made based on a TB ink film that was capable of measuring *P*_air_ values from 0.1 to 14 atm.^39^

#### 3.3.4 Dissolved CO_2_

The lipophilic, solvent-based CO_2_ indicator inks described in this section have also been used to measure the level of CO_2_ dissolved in water, although they tend to quickly (within 1 h) change permanently to the color of the protonated form of the dye, HD, when placed in acidic solution, or in ones that contain a high concentration (1 M) of a salt, such as NaCl. This change in color, and loss in function, is due to the anion, A^–^, in the acidic or salty solutions, exchanging with D^–^ in the ion-pair, Q^+^D^–^·*x*H_2_O in the film, to form Q^+^A^–^·*x*H_2_O, forcing D^–^ to form the lipophilic species HD;^40^ a transition in form and color that is effectively permanent as Q^+^A^–^·*x*H_2_O is very stable. The situation is improved by replacing the usual TOAH base with tetradodecyl ammonium hydroxide or tetrakisdecylammonium hydroxide;^41^ but for long-term use, CO_2_ indicator ink needs to be covered with a GPM.

## 4 CO_2_ Indicator Pigments and Plastic Films

Although a number of solvent-based CO_2_ indicator inks have been developed, they have not had much impact commercially, because of the printing ink industry change from solvent- to water-based inks. The commercial viability of CO_2_ indicators has been improved with the development of the CO_2_-sensitive plastic films, which are waterproof and can be produced cheaply using a scalable process. A typical CO_2_-sensitive plastic film comprises a CO_2_-sensitive pigment embedded in an inert, low melting point polymer film, such as low-density polyethylene, LDPE, by extruding a mixture of the pigment and polymer together. The pigment comprises nanoparticulate silica powder particles coated with a mixture of the dye and PTA,^42−44^ and so its formulation can be abbreviated to, dye/PTA/SiO_2_, and that of the final, extruded plastic film as dye/PTA/SiO_2_-LDPE. The preparation and characterization details for a MCP/TBAH/SiO_2_ pigment and MCP/TBAH/SiO_2_-LDPE film, along with those of an MCP/TBAH/EC/TBP ink film, are given in sections S2 and S6 of the SI. All three exhibit a similar sensitivity and so same, α, values, although the plastic film is slower in response and recovery. Reasons for the latter and further discussion of the differences between the ink and plastic film indicators are given in S6 in the SI. Note that the response of a plastic film indicator depends upon dye p*K*_a_, [Base], and temperature in the same way as its ink film counterpart, and as described in sections 3.1 and 3.2, respectively. Thus, to minimize repetition, only potential applications of plastic films are considered below.

### 4.1 Applications

CO_2_-sensitive, plastic film indicators can be used to replace the applications identified in section 3.3 for their ink film counterparts. However, one of their most striking features is its ability to function in very wet, possibly highly saline, environments, such as found in seawater, food packaging (of meats, say), and wounds. Thus, examples of their use in such fields are given below.

#### 4.1.1 Plastic Films for Measuring Dissolved CO_2_

**Figure 7:**
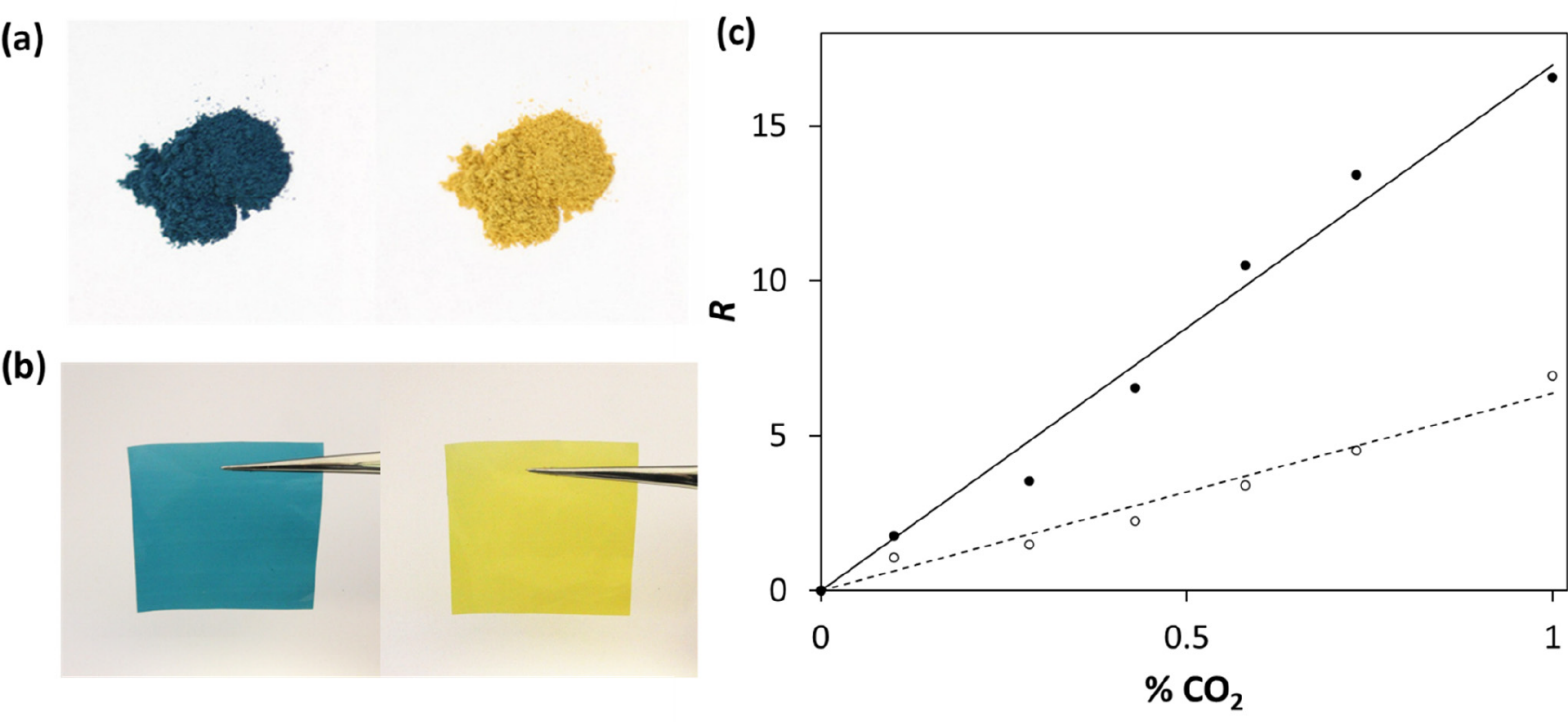
Photographs of (a) the silica pigment, coated with TB/TBAH, and (b) the final, extruded CO_2_-sensitive plastic film indicator, before (blue) and after (yellow) exposure to 100% CO_2_; (c) plots of *R* vs % CO_2_ generated using the TB plastic film in the gas phase (solid data points and line) or in a 0.6 M NaCl aqueous solution (open circle data points and broken line); the gradients of these two lines are 15.9 and 5.6%^–1^, respectively. [TB/TBAH/SiO_2_-LDPE, *T* = 20 °C; *f* = 100 cm^3^/min.] Adapted with permission from ref (43). Copyright 2016 Elsevier.

As noted earlier, bare (no GPM) CO_2_ inks cannot be used to measure % CO_2_ in a highly saline aqueous solution. In contrast, plastic film indicators are stable in even high ionic solutions, such as seawater, and this feature was illustrated using a TB plastic film indicator.^43^ Photographs of the TB/TBAH/SiO_2_ pigment and the final, extruded TB plastic film, TB/TBAH/SiO_2_-LDPE, before (blue) and after (yellow) exposure to 100% CO_2_ are illustrated in Figure 7a and b, respectively.^43^ The absorbance, *A*, of the CO_2_-sensitive TB plastic film was measured as a function of % CO_2_, both in the gas phase and in a 0.6 M NaCl aqueous solution, and the *A* vs % CO_2_ data were then used to construct the *R* vs % CO_2_ plots illustrated in Figure 7c. From the latter plots, it is clear that the sensitivity of the TB plastic film is reduced significantly (by a factor of ca. 2.8) when used to measure % CO_2_ in saline solution compared to that in a gas, with *P*_CO2_(*S* = 1/2) = 0.18% and 0.063% in saline solution and the gas phase, respectively.^43^

Other work shows that the loss of sensitivity that accompanies using a plastic film to measure % CO_2_ in water rather than the gas phase is a common feature for all plastic film indicators and appears to be due to the reversible uptake of water by the plastic indicator films. However, most importantly, unlike its ink counterpart, the TB CO_2_-sensitive plastic film indicator is indefinitely stable in salty aqueous solutions.^43^ This initial (but reversible) loss of sensitivity exhibited by plastic films when used for dissolved CO_2_ measurements is due to an associated drop in the solubility of CO_2_ in the encapsulation medium, which decreases with increasing polarity and hydrogen-bonding character of the solvent/encapsulation medium. Indeed, in aqueous solution, the sensitivity of the TB CO_2_ plastic film indicator is very similar to that of a water-based ink based on the same dye, TB/TBAH/PVA, but operating in the gas phase.

#### 4.1.2 After Opening Freshness (AOF) Indicator

**Figure 8:**
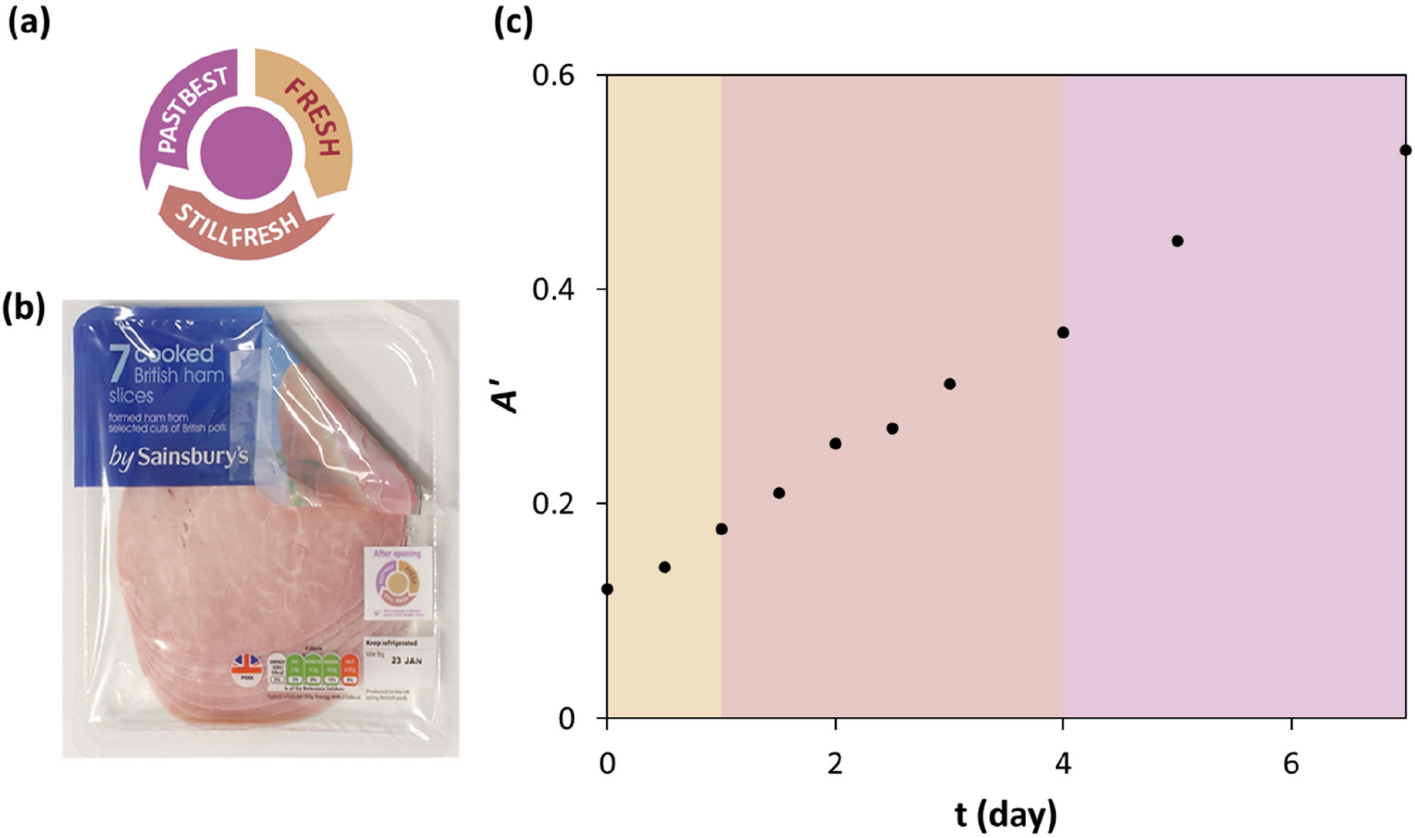
Photographs of (a) the AOF label and (b) the label in the refrigerator food package of ham; (c) plot of apparent absorbance, *A’*, vs time after opening for an AOF label. [CR/TBAH/SiO_2_-LDPE, *T* = 5 °C; *f* = 0 cm^3^/min.] Adapted with permission from ref (46). Copyright 2018 Elsevier.

Insignia Technologies Ltd. have recently developed an “After Opening Freshness”, AOF, label, based on the CO_2_-sensitive plastic film technology developed by the Mills group,^44^ that is able to inform the consumer whether the food in an opened food package in the fridge is still fresh, and so safe to eat.^45^ Photographs showing the label and how it is incorporated into a typical refrigerator food package are illustrated in Figure 8a and b, respectively. Research shows that the AOF label illustrated in Figure 8 comprises a CO_2_-sensitive CR plastic film sandwiched between two polymer layers, one of which is a barrier layer that controls the rate of permeation of CO_2_ from inside the CR film to outside the label. A detailed schematic illustration of the structure of the label is illustrated in section S7, Figure S5 of the SI.

The AOF indicator is effective when used with modified atmosphere food packages, MAP, since the high level of CO_2_, typically >20%, usually used in MAP, is sufficient to change the color of the CR indicator film in the AOF label from purple, its color in air, to beige. In a MAP sealed package of ham, with % CO_2_ = 25%, the CR indicator in the AOF label is initially beige but changes color when the package is opened, since the ambient level of CO_2_ then drops to 0.04%. Under these circumstances, in the absence of the barrier film covering, the CR plastic film would normally regain its original (purple) color within ca. 26 min; but when incorporated in the AOF label, a diffusion-barrier film cover layer of 30 μm polyethylene terephthalate, PET, slows the rate of CO_2_ permeation from inside to outside the label to such an extent that, at 5 °C, the typical temperature of a household fridge, the CR plastic film in the label takes about 4 days to turn purple. As illustrated in Figure 8a, in the AOF indicator, the color change of the central CR indicator “dot”, from beige to purple, is taken as an indication that the food in the opened package is “past best”.^46^ Other work shows that it is possible to analyze the color of a photograph of the CO_2_ indicator to derive a value for its apparent absorbance, *A*’, that is directly proportional to its real absorbance, *A*; this process is referred to here as digital camera colorimetry, DCC.^47^ Further details of DCC and its use with CO_2_ indicators are given in section S8 in the SI. Thus, Figure 8c shows how *A’*, for the AOF label, varies as a function of after opening time and highlights the time periods when the label is in its 3 different colored forms, and so signaling, “fresh” (beige), “still fresh” (brown), and “past best” (purple).

#### 4.1.3 Early Wound Infection Indicator

Chronic wounds require careful tending and continuous monitoring, and in the UK alone, the annual cost of managing chronic wounds is over £3.2 billion. Unfortunately, by the time signs of infection appear in a chronic wound, the infection has often taken hold. Interestingly, there is strong evidence that infection is associated with surpassing a critical colony threshold, CCT, of ca. 10^6^ colony forming units per gram of tissue, i.e., CFU g^–1^, regardless of the infecting species. Unfortunately, for reasons of cost, the measurement of microbial load is not a routine part of wound monitoring.

Currently, most current chronic wound dressings are occlusive, i.e. sealed, so as to reduce the chance of infection from outside,^48^ but, since such a dressing seals in the headspace above a wound, it follows that a CO_2_-indicator could be used to identify any appreciable increase in CO_2_, above the ambient value of ca. 0.04%, which might be expected if the wound were infected.

**Figure 9:**
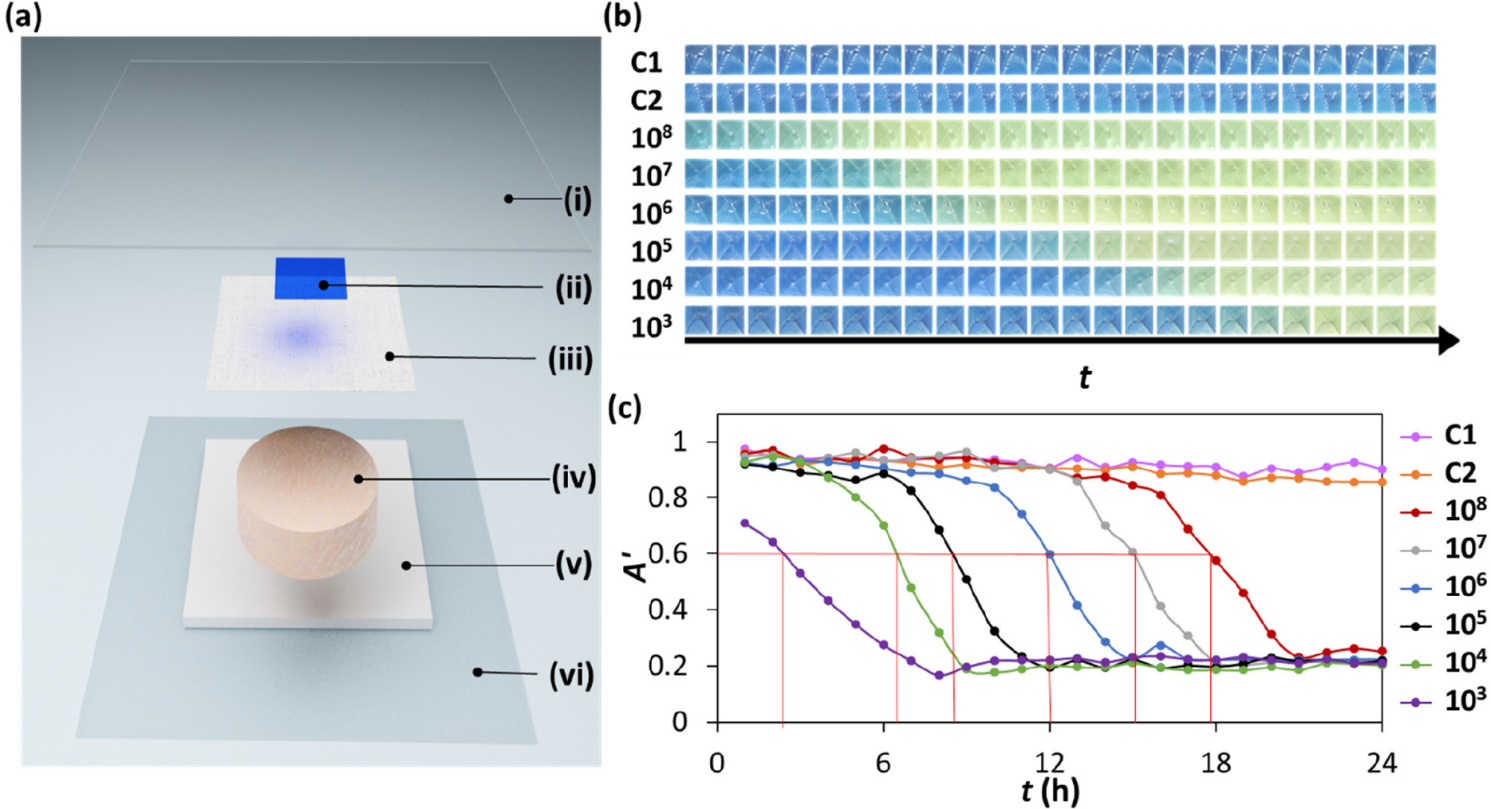
(a) Schematic illustration of the set up used to test a XB plastic film as an early wound infection indicator, comprising (i) clear wound dressing plastic adhesive film, (ii) XB plastic film CO_2_ indicator, (iii) gauze, (iv) infected pig-skin, (v) wet absorbent pad, and (vi) inert support substrate (50 μ PET); (b) photographs of the XB film in (a), and (c) measured variations in *A’*, determined from the images in (b), as a function of incubation time, *t*, for the pigskin inoculated with different loadings of *Pseudomonas aeruginosa* (CFU mL^–1^). [XB/TBAH/SiO_2_-LDPE, *T* = 30 °C; *f* = 0 cm^3^/min.] Adapted with permission from ref (48). Copyright 2022 Royal Society of Chemistry.

Recently, a noninvasive, inexpensive, easy to use early wound-infection indicator, based on a 3D printed, CO_2_ plastic film, has been reported which signals when the microbial load associated with a wound approaches 10^6^ CFU g^–1^.^48^ The indicator used a xylenol blue, XB, plastic film and was tested using an infected wound model based on pig skin, a schematic of which is illustrated in Figure 9a. The color of the XB plastic film indicator was then monitored, via photography, as a function of *t*, for different initial inoculum loadings, and the results of a typical set of runs are illustrated in Figure 9b, which show, not surprisingly, that the higher the initial level of the inoculum, the faster the production of CO_2_ in the headspace. The photographs associated with runs C1 and C2 illustrated in Figure 9b also show that the indicator did NOT change when the wound bed was not inoculated (run C1) or if undamaged (no lacerations) pigskin was inoculated (run C2).^48^

From each of the *A*’ vs *t* profiles illustrated in Figure 9c, the time taken for *A’* to fall to a value of 0.6 was determined, *t*(*A*’ = 0.6). In a separate but otherwise identical set of experiments to those used to generate the data in Figure 9, for each inoculum, the value of the total microbial load on the pigskin, units CFU g^–1^, was measured as a function of *t*, from which it was possible to determine the time taken for the load to reach the CCT value of 10^6^ CFU g^–1^, *t*(10^6^). The subsequent plot of *t*(*A*’ = 0.6) vs *t*(10^6^) yielded a good straight line with a near unity gradient, suggesting that the XB plastic film can be used to signal when the bacterial load on the wound is at or near ca. 10^6^ CFU g^–1^.^48^ Other work showed that the XB indicator worked equally well with other anaerobes associated with wound infection, such as *Enterococcus. faecium, Acinetobacter baumannii, Streptococcus pyogenes, Candida albicans, and Staphylococcus aureus.*

#### 4.1.4 A CO_2_ Indicator Adhesive

Recently, it has been demonstrated that a CO_2_-sensitive pigment can be easily incorporated into a pressure sensitive adhesive, PSA.^49^ Such a smart adhesive is simple to make and can be readily applied to a plastic film to make very inexpensive CO_2_-sensitive labels or tape.

## 5 Outlook

A brief comparison between the four different CO_2_ indicator types reported here, namely, aqueous, ink, plastic, and adhesive films, is given in section S9, Table S4 in the SI. From this table, the outlook of the CO_2_ sensitive plastic film and adhesive indicators appears particularly promising, especially when coupled with DCC,^47^ which can be carried out with just a mobile phone and app. Although most work on CO_2_ indicators is focused on their use in food packaging, this Account shows they have potential to be used in many different areas, such as in capnography, thermometry, manometry, wound monitoring, and environmental monitoring.

## Supporting Information Available

Table of previous landmark papers, ink, pigment and plastic film indicator formulations, details on measuring response/recovery times, table of dyes used, details of a CO_2_ indicator based temperature probe, comparison of pigment, ink and plastic film indicators, AOF indicator schematic, outline of DCC applied to CO_2_ indicators and table comparing the characteristics of all 4 indicator types (PDF)
