# Supplementary material for: Colorimetric CO2 Indicators
Source: Acc Mater Res. 2023 Jun 19;4(7):570–9. doi: 10.1021/accountsmr.2c00226 (PMC10391618; doi:10.1021/accountsmr.2c00226)
Supplement: Supplementary file 1 — mr2c00226_si_001.pdf [file mr2c00226_si_001.pdf]

Electronic Supplementary Information

(ESI)

Colorimetric CO_2 indicators

Andrew Mills, Lauren MacDonnell and Dilidaer Yusufu*

School of Chemistry and Chemical Engineering, Queens University Belfast, David Keir Building, Stranmillis Road, Belfast, UK, BT95AG

e-mail: andrew.mills@qub.ac.uk

S1. Key reports on colorimetric CO_2 indicators

Table S1. Key reports on colorimetric CO_2 indicators

Indicator type	Dye[a]	Medium[b] (solvent)	Base[c]	Comment	Ref
Aqueous solution	PP and CR	(Water)	$NaHCO_3$	Early method of confirming correct endotracheal intubation in hospitals using pH sensitive dye in solution (no GPM).	1
	BTB	RTIL (water)	TOAH	The first example of a room temperature ionic liquid, RTIL, has been used as matrix material in the optical CO_2 sensor design (no GPM).	2
	PR or BTB	(Water)	NaOH	One of the early methods of colourimetric measurement of pCO_2 in seawater (GPM: silicone membrane).	3
Ink film	CR	Silicone rubber	TOAH	An early example of indicator anion and quaternary ammonium cation dissolved in silicone rubber which makes the CO_2 indicator applicable to optical sensing of both dry gases and aqueous solutions.	4
	BTB and MR	MC (water)	$NaHCO_3$	An early example of colourimetric *water-based* CO_2 ink film.	5
	BTB or PR	poly(HEMA) hydrogel (water)	$NaHCO_3$	The first example of fluorescence resonance energy transfer, FRET, based sensor in which the acceptor pH sensitive dye (BTB or PR) causes the lifetime changes of the doner fluorescent dyes (Eosin, R6G, or TRH) (GPM: silicone membrane).	6
	NAF	EC (toluene)	TOAH	An early example of an optical CO_2 sensor ink film which is based on fluorescence intensity changes (TPP) due to the absorption change of a pH sensitive dye (NAF).	7

[a]: PP: phenolphthalein; CR: cresol red; BTB: bromothymol blue; PR: phenol red; MR: methyl red; NAF: α-naphtholphthalein; R6G: Rhodamine 6G, TRH: Texas red hydrazide; TPP: tetraphenylporphyrin; [b]: RTIL: room temperature ionic liquid; EC: ethyl cellulose; MC: methylcellulose; poly(HEMA): Polyhydroxyethylmethacrylate; [c]: TOAH: tetraoctylammonium hydroxide; GPM: gas permeable membrane; FRET: fluorescence resonance energy transfer.

References

[1] Berman, J.; Furgiuele, J.; Marx, G. The Einstein carbon dioxide detector. *Anesthesiology* **1984**, *60* (6), 613-614.

[2] Oter, O.; Ertekin, K.; Topkaya, D.; Alp, S. Room temperature ionic liquids as optical sensor matrix materials for gaseous and dissolved CO2. *Sensors and Actuators B: Chemical* **2006**, *117* (1), 295-301.

[3] DeGrandpre, M. D. Measurement of seawater pCO_2 using a renewable-reagent fiber optic sensor with colorimetric detection. *Anal. Chem.* **1993**, *65* (4), 331-337.

[4] Weigl, B. H.; Wolfbeis, O. S. New hydrophobic materials for optical carbon dioxide sensors based on ion pairing. *Anal. chim. acta* **1995**, *302* (2-3), 249-254.

[5] Chen, H.-z.; Zhang, M.; Bhandari, B.; Guo, Z. Applicability of a colorimetric indicator label for monitoring freshness of fresh-cut green bell pepper. *Postharvest Bio. Technol.* **2018**, *140*, 85-92.

[6] Lakowicz, J. R.; Szmacinski, H.; Karakelle, M. Optical sensing of pH and pCO_2 using phase-modulation fluorimetry and resonance energy transfer. *Anal. chim. acta* **1993**, *272* (2), 179-186.

[7] Amao, Y.; Nakamura, N. Optical CO_2 sensor with the combination of colorimetric change of α-naphtholphthalein and internal reference fluorescent porphyrin dye. *Sensors and Actuators B: Chemical* **2004**, *100* (3), 347-351.

S2. Preparation of different CO_2 indicators

S2.1 A CO_2-sensitive MCP ink film indicator

The preparation of a typical CO_2 sensitive ink film has been described elsewhere [1], but briefly, 0.012 g metacresol purple (MCP) were dissolved in 2.5 ml methanol and 1 ml of 0.5 mol dm^{-3} tetraoctylammonium hydroxide (TOAH) in methanol was further added to the solution. 1 ml of this solution was mixed with 10 g of ethyl cellulose (EC) solution, comprising 10 wt% of EC dissolved in a solvent of 20 ml ethanol and 80 ml toluene), to which 1 ml of the plastisizer, tributyl phosphate (TBP) and 1 ml of the 0.5 M TOAH methanol solution had also been added. The final mixture was stirred with magnetic stirrer for 1 hour. The dry MCP ink film was generated by spin coating 1.5 ml of the MCP ink on a microscope slides with a spin speed of 1500 rpm for 15 s. Photographs of the final spin coated MCP ink film under ambient air and 100 %CO_2 were given in Figure S1 below.

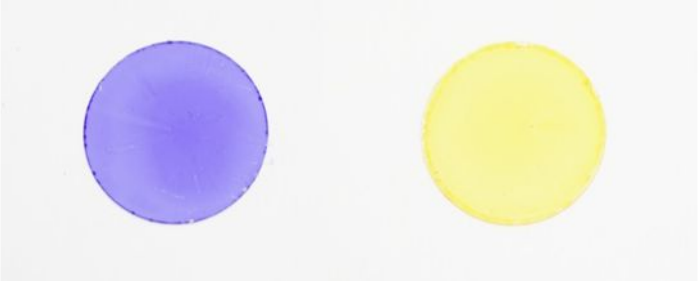

Figure S1: Spin coated MCP ink film on a microscope slide under ambient air (left) and 100 %CO_2 (right).

The purple to yellow color change exhibited by the CO_2-sensitive, MCP ink film indicator upon exposure to CO_2 was completely reversible.

S2.2. A MCP-based, CO_2 sensitive pigment

Making of a typical CO_2 sensitive pigment is described elsewhere [2, 3]. In this work, briefly, a CO_2-sensitive MCP pigment was made by mixing (i) 0.2 g MCP, 3.1 ml tetrabutylammonium hydroxide, TBAH, 1M, in methanol and (ii) 2 g of hydrophilic fumed silica (Aerosil 130 V, particle size ~20 nm) in 100 ml ethanol in a 200 ml glass beaker. The suspension was then stirred vigorously for 2 hours using a magnetic stirrer, and the solvent removed by rotary evaporation to yield a dry, blue-coloured pigment comprised of a coating of MCP/TBAH on hydrophilic silica particles. Photographs of the CO_2 sensitive MCP pigment before and after exposure to 100 %CO_2 is given in Figure S2 below.

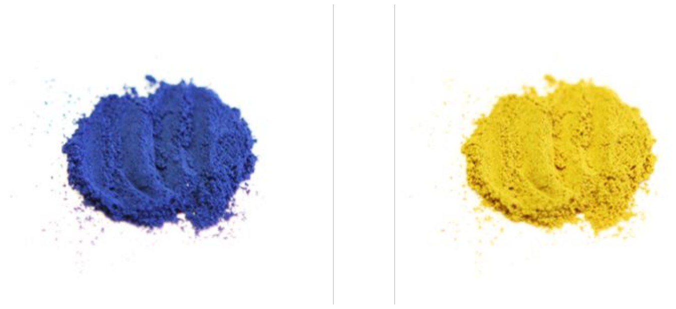

Figure S2: Photograph of a CO_2 sensitive MCP pigment under ambient air (left) and in 100% CO_2 (right).

S2.3. A CO_2 sensitive MCP plastic film

A CO_2-sensitive MCP plastic film was fabricated using a procedure that has been reported elsewhere [2,3], *via* the extrusion of a mixture of the MCP pigment, the preparation of which is described in S1.3, with low-density polyethylene (LDPE) powder. Thus, 2 g MCP pigment and 18 g LDPE powder (melt flow index, MFI, 20) were mixed and fed through a Rondol Microlab 10 mm twin screw extruder (barrel L/D 25/1), with a processing temperature that increased gradually from 90 °C at the feed zone, to 140 °C at the die to produce 10 wt% MCP pigmented LDPE master batch. The extruder screw speed was maintained at 80 rpm, and feed hopper rate at 41 rpm. The resulting 2 mm diameter master batch strand was then fed into an on-line pelletizer to create 3 mm long pellets.

The master batch pellets were then 'diluted' by 50% w/w using the supplied LDPE pellets (MFI 4), to produce the final 5 wt% MCP pigment in LDPE loading required for the final extruded CO_2-senstive MCP plastic film. The thermal processing conditions for producing the MCP plastic film were: 90 °C (at the feed zone), increasing to 110–125–135 °C (across the barrel) and finally 140 °C (at the die zone). The feed hopper rate was 20 rpm, the extruder screw speed was 80 rpm, and the extruded film take-off speed was 1.7 m min^{-1}. The final plastic film product, a 5 wt% pigmented MCP-LDPE film, ca. 50 ± 3 μm thick, was blue-colored in the absence of CO_2, but rapidly turned yellow upon exposure to a stream of CO_2 (see Figure S3below).

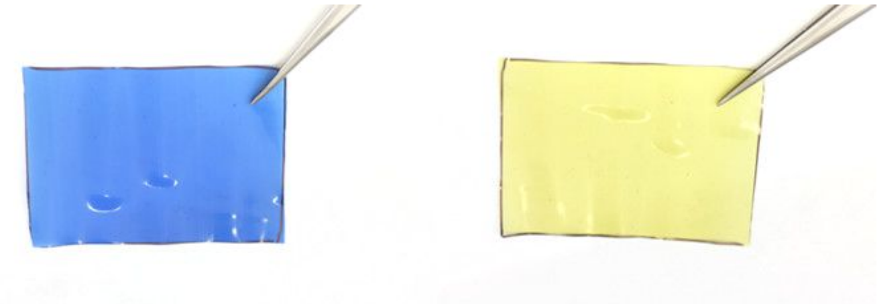

Figure S3: Photograph of CO_2 sensitive MCP plastic film under ambient air (left) and 100 %CO_2 (right).

References

[1] Mills, A., Qing, C. and Neil, M., Equilibrium studies on colorimetric plastic film sensors for carbon dioxide, *Anal. Chem.*, **1992**, 64, 1383-1389.

[2] Mills, A. and Yusufu, D. Highly CO_2 sensitive extruded fluorescent plastic indicator film based on HPTS, *Analyst*, **2016**, 141, 999-1008.

[3] Mills, A. and Yusufu, D., Extruded colour-based plastic film for the measurement of dissolved CO_2, *Sensors and Actuators B*, **2016**, 237, 1076-1084.

S3. Measurement of the typical response characteristics of the different CO_2 indicators

Each of the indicators prepared in S2 was characterized in terms of their response characteristics. In each case, the UV/Vis absorbance spectrum of the indicator was recorded as a function of % CO_2, the level of which was varied using a gas blender. From these spectra the absorbance of the deprotonated form of MCP, typically at 593 nm, i.e. A, was determined and the following equation used to determine the value of R. In each case, and in accordance with eqn S1, which is the same as eqn (5) in main text, a plot of R vs %CO_2 yielded a good straight line of gradient, α.

$$(A_o - A)/(A - A_\infty) = R = \alpha P_{CO2} \quad \text{(S1)}$$

In a separate study the absorbance of the indicator was measured in an atmosphere which was varied between 0 and 100 %CO_2 and from the resulting A vs time profiles, the times taken for the indicator to complete 90% of its change in color in response to 100 %CO_2 (response time, $t_{90}\downarrow$) and Ar (recovery time, $t_{90}\uparrow$) were determined.

S4. Different pH indicator dyes used to make CO_2 indicators

Table S2. Names, abbreviations, pK_a's and colors of different pH indicator dyes used to make CO_2 indicators

**Name**	**Abbreviation**	**pKa†,*, ††**	**Colour change (D^- to DH)**
Xylenol blue	XB	9.52†	Blue to yellow
Phenolphthalein	PP	9.46*	Violet to colourless
o-Cresolphthalein	OCP	9.32*	Red to colourless
Thymol blue	TB	8.86*	Blue to yellow
m-Cresol purple	MCP	8.28*	Blue to yellow
Cresol red	CR	7.95*	Purple to yellow
Phenol red	PR	7.52*	Purple to yellow
Rosolic acid	RA	7.40*	Red to yellow
Bromothymol blue	BTB	7.30††	Blue to yellow

† Taken from [1]. *Taken from [2]. †† Taken from own experimental work.

References

[1] Yusufu, D.; Magee, E.; Gilmore, B.; Mills, A. Non-invasive, 3D printed, colourimetric, early wound-infection indicator. *Chem. Commun.* **2022**, *58*, 439-442.

[2] Mills, A.; Chang, Q. Tuning colourimetric and fluorimetric gas sensors for carbon dioxide. *Anal. Chim. Acta* **1994**, *285*, 113-123.

S5. Using a CO_2 ink film as a temperature probe

As noted in section 3.2 of the main text, given their temperature sensitivity, a CO_2 indicator film could be used as a temperature indicator, if the value of P_{CO2} was set at some fixed value, P_{CO2}(fxd). And, indeed, a temperature indicator based on a PP CO_2-sensitive ink film has been made by using the CO_2 indicator in air, where P_{CO2}(fxd) is *ca.* 0.04% [1]. A typical set of photographs of the indicator, a PP ink film, with increasing T, and a subsequent plot of its absorbance, *A*, vs T plot, both recorded for in ambient air, are illustrated in Figures S4(a) and (b), respectively [1].

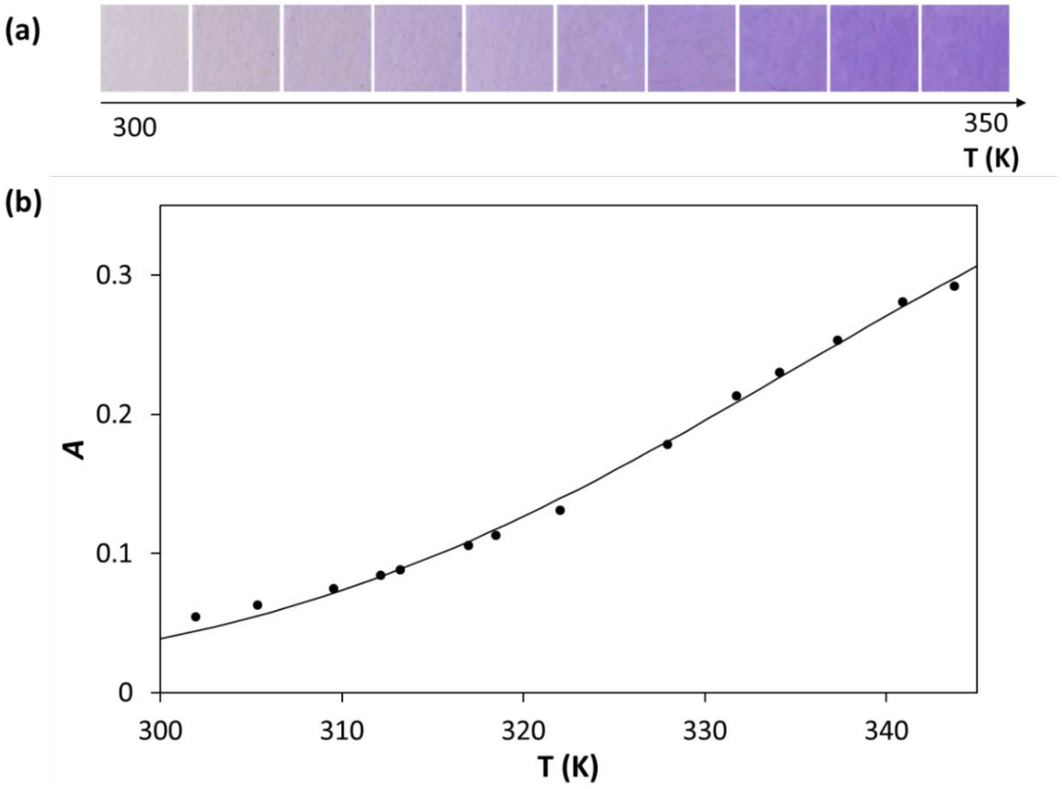

Figure S4. (a) Photographs of a PP ink film as a function of temperature and (b) subsequent plot of the absorbance of the film, *A,* (at λ(max) for D^-) as a function of film temperature. [PP/EC/TOAH/TBP films; T = 20 ^oC; *f* = 0 cm^3/min, RH = 0%]. Adapted with permission from ref [1]. Copyright 1999 Royal Society of Chemistry.

The solid line is the best fit to the data based on a theory-based equation. Other such colorimetric temperature sensors, using OCP and TB, were generated for probing different temperature ranges [1].

References

[1] Mills, A.; Lepre, A. Development of novel thermochromic plastic films for optical temperature sensing. *Analyst* **1999**, 124, 685-689.

S6. CO_2-sensitive, inks and plastic films – a comparison

A typical CO_2-sensitive plastic film comprises a CO_2-sensitive pigment embedded in an inert, low melting point, polymer film, such as LDPE, by extruding a mixture of the pigment and polymer together. The pigment comprises nanoparticulate silica powder particles coated with a mixture of the dye and PTA [1, 2], and so its formulation can be abbreviated to, dye/PTA/SiO_2, and that of the final, extruded plastic film as dye/PTA/SiO_2-LDPE. Details of how a typical CO_2-sensitive pigment, based on the dye MCP, is prepared are given in S2.2 and details of how this pigment, MCP/TBAH/SiO_2, is then mixed with low-density polyethylene, LDPE, and extruded to produce a CO_2-sensitive plastic film, MCP/TBAH/SiO_2-LDPE are given in S2.3. In the production of CO_2-snestive plastic films, LDPE is usually chosen as the extrusion polymer because of its inert, non-acidic nature, high CO_2 permeability and low processing temperature (melting point: 85-125 ^oC [3]). A low processing temperature is required as the indicator ink that coats the silica particles decomposes above 180 ^oC.

A brief comparison of the measured sensitivities (α) and $t_{90}{\downarrow}$ and $t_{90}{\uparrow}$ times for a typical MCP-based, CO_2-sensitive, (i) ink film, (ii) pigment and (iii) plastic film are given in Table 1. Further details as to how the values of the parameters given in Table 1 were measured are given in section S3 in the ESI.

Table S3. Measured sensitivities and response and recovery times for a CO_2-sensitive MCP-based ink, pigment and plastic film

CO_2 indicators (polymer/PTA)	α (%$^{-1}$)	$t_{90}{\downarrow}$ (s)	$t_{90}{\uparrow}$ (s)	thickness (μm)
MCP ink film (EC/TOAH)*	0.78 ± 0.02* (3.57)§	2.6*	31*	20*
MCP pigment (none/TBAH)	3.37 ± 0.09	3.2	254	-
MCP plastic film (LDPE/TBAH)	3.27 ± 0.098	10	1640	50

*: from [4]; §: when TOAH is replaced with tetra butyl ammonium hydroxide, TBAH. Note:

Table 1 shows that whereas the MCP ink is fast in response and recovery, the MCP plastic film is not, although the latter can be reduced significantly (factor of 9 for 15 μm film) by using a thinner film [2]. Although the MCP plastic film is slower to respond and recover than its ink film counterpart, it has distinct advantages which include a much higher stability when, (i) stored under ambient conditions and (ii) used in highly saline and acidic solution, even > 1 M [2]. This greater stability is due to the greater efficacy of the extruded, hydrophobic CO_2-indicator polymer to act as a waterproof, GPM, compared to the polymer, such as EC, used in the ink films. Consequently, ink films can only be used in highly saline or acidic solution if covered with a water-proof GPM, like PTFE. In plastic film indicators, the role of a GPM is to not only prevent ionic species, such as ion-exchange anions, A^-, access to the indicator ion pairs, but also to prevent the ion-pair being leached out, or losing the water in $Q^+D^-.xH_2O$, both of which leads to loss of function. The loss of water by the indicator ink film is also reduced in the plastic film due to the hygroscopic nature of the hydrophilic silica which supports the indicator in the extruded plastic films. The loss of water by the indicator ink film is also reduced in the plastic film due to the hygroscopic nature of the hydrophilic silica which supports the indicator in the extruded plastic films.

The other important feature of the plastic film indicator is its capacity for low cost, scaled mass production.

Further inspection of the results in Table S1 suggests that the low value of α for the MCP film appears inconsistent with those exhibited by the MCP pigment and plastic film. However, this is due to the use of a different phase transfer agent/base, TOAH, in the ink than that used to make the pigment and plastic film, namely TBAH. When TBAH was used to make a MCP ink film, the value of α was found to be ca. 3.57 % which is much more consistent with that found for the MCP pigment and plastic film. The improved sensitivity of the MCP ink film with TBAH, compared to TOAH, is due to the higher binding efficiency of quaternary cation, resulting in a bigger value for α for reaction (7) [5].

References

[1] Mills, A.; Skinner, G. A.; Grosshans, P. Intelligent pigments and plastics for CO_2 detection. *J. Mater. Chem.* **2010**, 20, 5008-5010.

[2] Mills, A.; Yusufu, D. Extruded colour-based plastic film for the measurement of dissolved CO_2. *Sensors and Actuators B: Chemical.* **2016**, 237, 1076-1084.

[3] Brandrup, J., Immergut, E.H., Grulke, E.A., Abe, A. and Bloch, D.R. eds., *Polymer handbook*. New York: Wiley. **1999**.

[4] Mills, A., Qing, C. and Neil, M., Equilibrium studies on colorimetric plastic film sensors for carbon dioxide, *Anal. Chem.* **1992**, 64, 1383-1389.

[5] Goga, S.T., Lebed, A.V. and Mchedlov-Petrossyan, N.O., Conductivity and dissociation constants of quaternary ammonium perchlorates and picrates in 4-methyl-pentan-2-one. *J. Chem. Eng. Data*, **2010**. 55(5), 1887-1892.

S7. Structure of the After Opening Freshness (AOF) Indicator

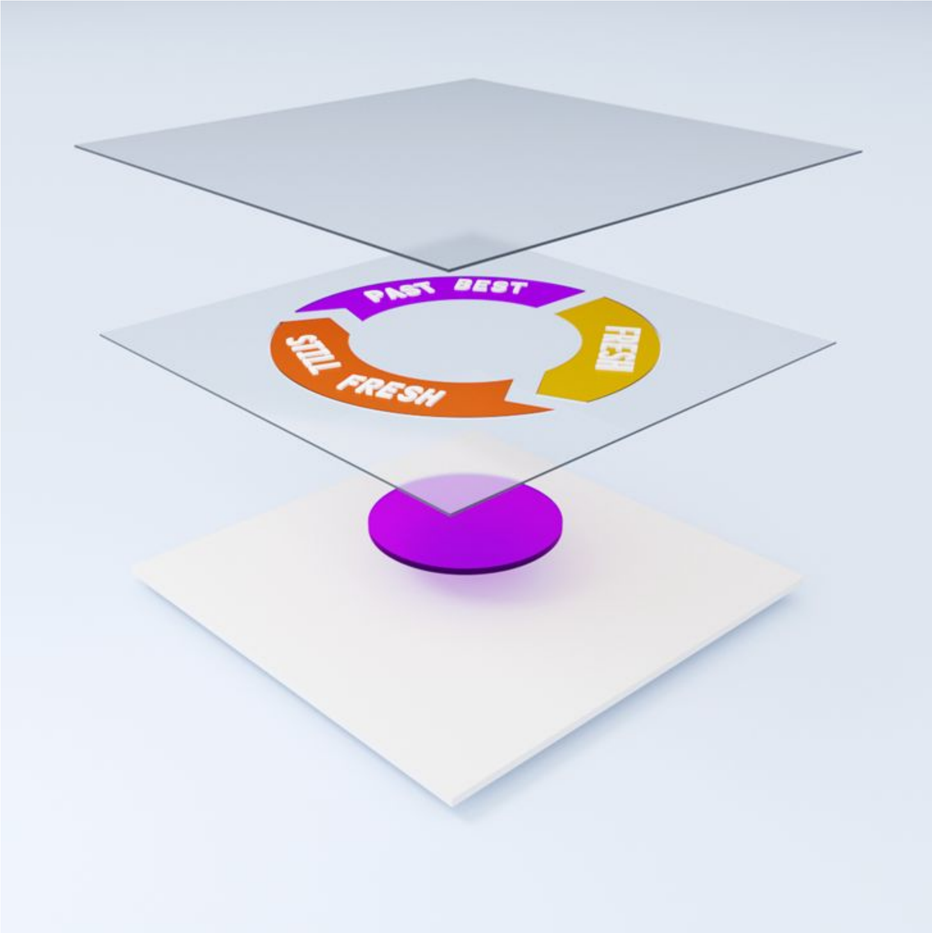

Figure S5. A typical AOF label attached to the clear package lidding comprised (from top to bottom), (i) the food package lidding layer made of clear PET with EVA coating (50 μm), (ii) a polypropylene, PP, barrier layer on which is printed reference colors (50 μm), (iii) a CR plastic film indicator layer (70 μm) and (iv) a CO_2 gas low permeation, barrier layer of white PET outer layer (30 μm).

S8. Digital Camera Colorimetry, DCC, and CO_2 indicators

The color of the digital photographic image of an indicator can be defined in terms of a device independent, standard Red (R), Green (G) and Blue (B) color space, RGB, where the values of R, G and B fall in the range zero to 255. There are many free software packages, such as Image J [1], that will generate the values of R, G and B associated with a digital image. In all the examples described in this account, the parameter that varies most significantly when the indicator changes color is R. It follows that for every digital image associated with an indicator, a value for apparent absorbance, *A'*, can be readily calculated, from the associated value of R, given,

$$A' = \log(255/R) \tag{S2}$$

In all the CO_2 indicator systems described in this account, and many other indicator systems [2], it is shown that A' is proportional to the real absorbance, A, due to the deprotonated form of the pH indicator dye in the CO_2 indicator film, at the wavelength it has its maximum absorbance. A simple demonstration of the direct relationship between *A'* and *A* can be obtained saturating an aqueous $NaHCO_3$, 1.43 mM, solution containing the pH indicator dye MCP with CO_2, so that it is converted from its initial red/purple deprotonated form to its yellow protonated form and then allowing it to lose its dissolved CO_2 to ambient air over time and so change slowly back from its yellow to its original (before saturation with CO_2) red/purple form.

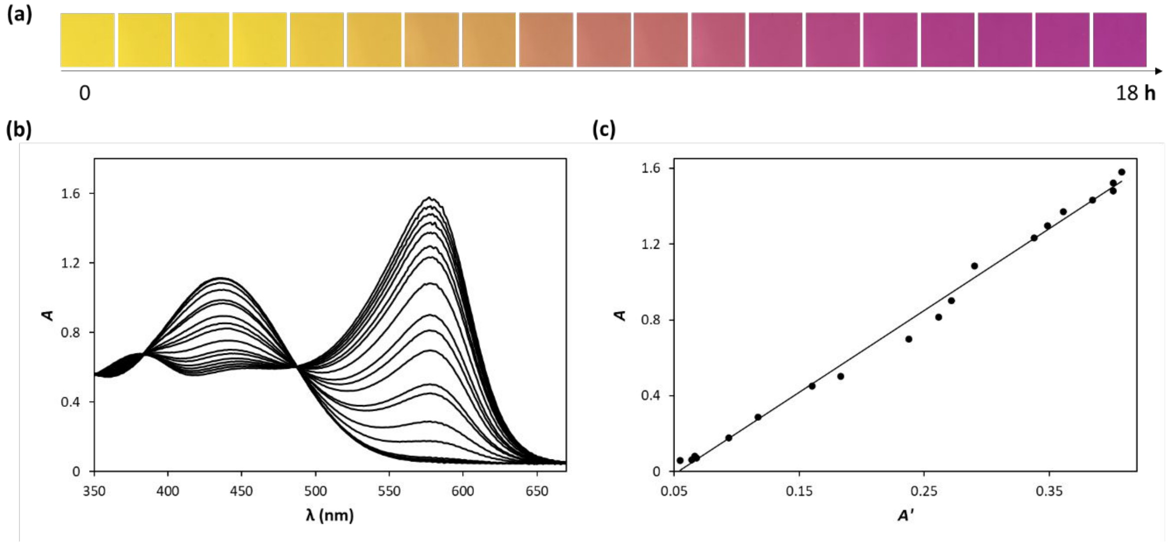

Figure S6. Regularly recorded, (a) digital photographic images and (b) UV/Vis absorption spectra of a $NaHCO_3$ aqueous solution (1.43 mM) containing the pH indicating dye MCP, initially saturated with 100% CO_2 for 10 minutes and then allowed to lose the dissolved CO_2 to ambient air over time and (c) subsequent plot of *A* vs *A'* for the same solution, with values of *A* and *A'* determined from the UV/Vis spectrum in (b) and from the photographic images in (a) using DCC, respectively.

Such a system was used to record both its color, and so value of *A*', using digital photography, and simultaneously its absorbance, *A, via* UV/Vis absorption, as the dye in the solution slowly changed from it protonated to deprotonated form. The results of this work, namely, Figure S6(a), the photographic images, Figure S6(b), the UV/Vis spectrum and Figure S6(c), the subsequent plot of *A* vs *A*' are illustrated below in Figure S6. The latter plot, Figure S6(c), provides a very good illustration of the direct relationship between *A* and *A*'. As a consequence, the *A* values in the key working equation S1, eqn (5) in the main text, can be readily replaced with their apparent absorbance, *A*', equivalents.

References

[1] Image J, https://imagej.nih.gov/ij/download.html, (accessed April 24, 2023).

[2] Yusufu, D.; Mills, A. Spectrophotometric and digital colour colourimetric (DCC) analysis of colour-based indicators. *Sensors and Actuators B: Chemical.* **2018**, 273, 1187-1194.

S9. CO_2-sensitive films of aqueous solution, ink, plastic and adhesive – a comparison

Table S4 Comparison between the four different types of CO_2 indicator

	**Aqueous**	**Ink film**	**Plastic film**	**Adhesive**
**Components**	Dye/base/GPM	Dye/polymer/PTA/plasticizer	Dye/PTA/SiO_2-LDPE	Dye/PTA/PSA
**Example**	BTB/$NaHCO_3$/PTFE [1]	MCP/EC/TOAH/TBP [2]	MCP/TBAH/SiO_2/LDPE [3]	PR/TBAH/tack Melt A43 [4]
**α (%$^{-1}$)**	2.3	0.75	3.27	0.61
**$t_{90}\downarrow$, $t_{90}\uparrow$ (s)**	1440, 6240	2.6, 31	10,1640	< 1, 2
**Inexpensive**	✓	✓	✓	✓
**Readily scaled**	×	✓*	✓	✓
**Printable**	×	✓*	✓	✓
**Suitable for use in wet environments**	Only with GPM	Only with GPM	✓	✓
**Shelf life**	years	weeks/months	years	years

*: But the manufacture and printing of solvent-based inks is increasingly rare due to health and safety reasons

The scalability and resistance to leaching and sensitivity loss in wet environments, such as found in highly saline and acidic solution, food packaging and wounds, make the plastic and, most recently, adhesive film technology the most commercially viable of the 4 indicator types.

References

[1] McDonnell, L., Yusufu, D., O'Rourke, C. and Mills, A., Enhancing the Performance of Two Different Commercial CO_2 Indicators Using Digital Colourimetric Analysis, DCA. *Chemosensors* **2022**, 10(12), p.544.

[2] Mills, A., Chang, Q. and McMurray, N. Equilibrium Studies on Colorimetric Plastic Film Sensors for Carbon Dioxide, *Anal. Chem.* **1992**, 64, 1383-1389.

[3] This work

[4] Wang, C.; Yusufu, D.; Mills, A. A smart adhesive 'consume within' (CW) indicator for food packaging. *Food Packag. Shelf Life* **2019**, 22, 100395.
